# Bis(μ-diethyl­phosphido-κ^2^ *P*:*P*)bis­[bis(2,4,6-trimethyl­phen­yl)indium(III)]

**DOI:** 10.1107/S1600536811042668

**Published:** 2011-10-22

**Authors:** Glen G. Briand, Andreas Decken, Dane A. Knackstedt, Caleb D. Martin

**Affiliations:** a Department of Chemistry and Biochemistry, Mount Allison University, 63C York Street, Sackville, New Brunswick, Canada E4L 1G8; b Department of Chemistry, University of New Brunswick, Fredericton, New Brunswick, Canada E3B 5A3

## Abstract

The title compound, [In_2_(C_9_H_11_)_4_(C_4_H_10_P)_2_], contains a centrosymmetric In_2_P_2_ core with short inter­molecular In—P bonds. This core has acute P—In—P and obtuse In—P—In bond angles compared with other [*R*_2_InP*R*′_2_]_2_ analogues, due to the presence of the bulky aromatic substituents on the In atom and the non-sterically demanding ethyl substituents on the P atom.

## Related literature

For related dimeric phosphanylindanes, see: Alcock *et al.* (1989 ▶); Wells *et al.* (1992 ▶); Aitchison *et al.* (1989 ▶); Beachley *et al.* (1987 ▶, 1993 ▶, 1995 ▶, 2001 ▶); Culp *et al.* (1997 ▶); Malik *et al.* (1996 ▶); Thomas *et al.* (2002 ▶); Wells *et al.* (1993 ▶); von Hanisch (2001 ▶). For related trimeric phosphanylindanes, see: Burns *et al.* (1994 ▶); Werner & Neumüller (1996 ▶); Banks *et al.* (1991 ▶).
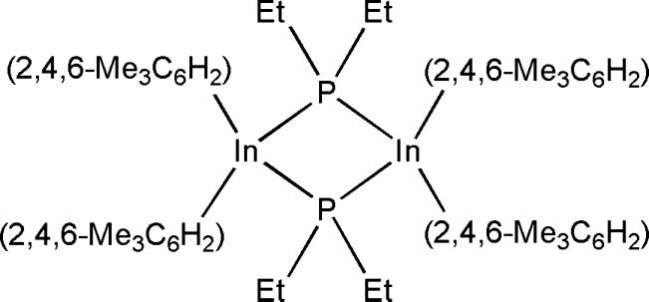


## Experimental

### 

#### Crystal data

[In_2_(C_9_H_11_)_4_(C_4_H_10_P)_2_]
*M* *_r_* = 884.53
Monoclinic, 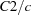

*a* = 22.323 (4) Å
*b* = 15.494 (4) Å
*c* = 14.331 (3) Å
β = 120.618 (4)°
*V* = 4265.6 (17) Å^3^
*Z* = 4
Mo *K*α radiation
μ = 1.18 mm^−1^
*T* = 198 K
0.23 × 0.20 × 0.01 mm

#### Data collection

Bruker SMART1000/*P*4 diffractometer
Absorption correction: multi-scan (*SADABS*; Sheldrick, 2008*a* ▶) *T*_min_ = 0.777, *T*_max_ = 0.988
14538 measured reflections
4771 independent reflections
3470 reflections with *I* > 2σ(*I*)
*R*_int_ = 0.038

#### Refinement

*R*[*F* ^2^ > 2σ(*F* ^2^)] = 0.031
*wR*(*F* ^2^) = 0.078
*S* = 1.09
4771 reflections
225 parameters
H-atom parameters constrained
Δρ_max_ = 0.65 e Å^−3^
Δρ_min_ = −0.34 e Å^−3^

### 

Data collection: *SMART* (Bruker, 1999 ▶); cell refinement: *SMART*; data reduction: *SAINT* (Bruker, 2006 ▶); program(s) used to solve structure: *SHELXS97* (Sheldrick, 2008*b* ▶); program(s) used to refine structure: *SHELXL97* (Sheldrick, 2008*b* ▶); molecular graphics: *DIAMOND* (Brandenburg, 2011 ▶); software used to prepare material for publication: *SHELXTL* (Sheldrick, 2008*b* ▶).

## Supplementary Material

Crystal structure: contains datablock(s) I, global. DOI: 10.1107/S1600536811042668/fj2456sup1.cif

Structure factors: contains datablock(s) I. DOI: 10.1107/S1600536811042668/fj2456Isup2.hkl

Additional supplementary materials:  crystallographic information; 3D view; checkCIF report

## Figures and Tables

**Fig. 1.**
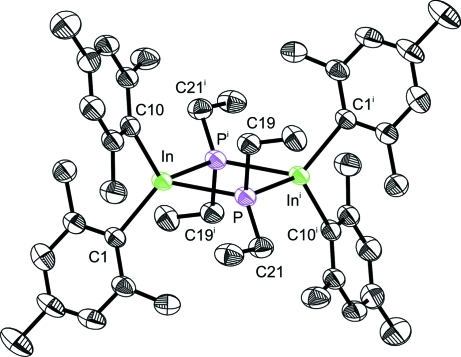
X-ray crystal structure of (I), with displacement ellipsoids drawn at the 50% probability level. H atoms have been omitted for clarity. Symmetry transformations used to generate equivalent atoms: (i) -x + 1/2, -y + 1/2, -z + 1. Selected bond distances (Å) and angles (°): In—P 2.6300 (12), In—Pi 2.6364 (9), In—C1 2.190 (3), In—C10 2.215 (3), P—C19 1.866 (3), P—C21 1.856 (3), P—In—Pi 81.56 (3), In—P—Ini 98.44 (3), C1—In—C10 117.6 (1), C19—P—C21 104.4 (2).

## supplementary crystallographic information

### Comment

Phosphanylindanes [*R*_2_InP*R*'_2_]_n_ form dimeric or trimeric structures in the solid state *via* intermolecular In—P coordinate bonding interactions (Beachley *et al.*, 2001; Werner & Neumuller 1996). Dimeric structures feature distorted tetrahedral geometries at indium and phosphorus, and planar four-membered In_2_P_2_ ring cores. In—P bond distances within the ring are similar and differ by less than 0.05 Å in all reported structures (Wells *et al.*, 1993). The structure of I (Fig. 1) shows a dimer in the solid state, with a characteristic In_2_P_2_ core and distorted tetrahedral geometries at the four coordinate indium and phosphorus centres. The In—P bond distances are similar [In—P = 2.6300 (12) Å, In—P^i^ = 2.6364 (9) Å] and are in the range for previously reported dimeric phosphanylindanes [2.612 (1)–2.712 (1) Å] (Wells *et al.*, 1993; Beachley *et al.*, 1993). However, the ring P—In—P^i^ bond angle [81.56 (3)°] is at the lower limit of the range of reported values for [*R*_2_InP*R*'_2_]_2_ structures [81.80 (7)–87.53 (3)°] (Beachley *et al.*, 1987; von Hanisch, 2001), and the In—P—In^i^ bond angle [98.44 (3)°] is at the upper limit of reported values [92.47 (3)–98.20 (7)°] (von Hanisch, 2001; Beachley *et al.*, 1987). The significant distortion of the In_2_P_2_ ring is likely a result of the bulky mesityl groups on indium, which affect a compression of the P—In—P^i^ bond angles. Conversely, the non-bulky ethyl groups on phosphorus allows for expansion of the In—P—In^i^ bond angles.

### Experimental

Preparation of [(2,4,6-Me_3_C_6_H_2_)_2_InPEt_2_]_2_ (I). Trismesityl indium (0.520 g, 1.10 mmol) was added to a solution of diethyl phosphine (0.100 g, 1.10 mmol) in toluene (7.5 ml). The reaction mixture was stirred at 45°C for 72 h, after which time a white precipitate had formed. The precipitate was removed by filtration, dried *in vacuo*, and washed with toluene (2 × 5 ml) and hexane (3 ml) (yield 0.110 g, 22%). Mp: 188°C. Crystals of I were obtained by dissolving the product in dichloromethane and allowing the solution to sit at 23°C for 12 h.

### Refinement

H atoms were included in calculated positions and refined using a riding model.

### Crystal data

**Table:**

[In_2_(C_9_H_11_)_4_(C_4_H_10_P)_2_]	*F*(000) = 1824
*M**_r_* = 884.53	*D*_x_ = 1.377 Mg m^−^^3^
Monoclinic, *C*2/*c*	Mo *K*α radiation, λ = 0.71073 Å
Hall symbol: -C 2yc	Cell parameters from 6933 reflections
*a* = 22.323 (4) Å	θ = 2.7–28.1°
*b* = 15.494 (4) Å	µ = 1.18 mm^−^^1^
*c* = 14.331 (3) Å	*T* = 198 K
β = 120.618 (4)°	Plate, colourless
*V* = 4265.6 (17) Å^3^	0.23 × 0.20 × 0.01 mm
*Z* = 4	

### Data collection

**Table:**

Bruker SMART1000/P4 diffractometer	4771 independent reflections
Radiation source: fine-focus sealed tube, K760	3470 reflections with *I* > 2σ(*I*)
graphite	*R*_int_ = 0.038
φ and ω scans	θ_max_ = 27.5°, θ_min_ = 1.7°
Absorption correction: multi-scan (*SADABS*; Sheldrick, 2008*a*)	*h* = −28→27
*T*_min_ = 0.777, *T*_max_ = 0.988	*k* = −19→19
14538 measured reflections	*l* = −18→18

### Refinement

**Table:**

Refinement on *F*^2^	Primary atom site location: structure-invariant direct methods
Least-squares matrix: full	Secondary atom site location: difference Fourier map
*R*[*F*^2^ > 2σ(*F*^2^)] = 0.031	Hydrogen site location: inferred from neighbouring sites
*wR*(*F*^2^) = 0.078	H-atom parameters constrained
*S* = 1.09	*w* = 1/[σ^2^(*F*_o_^2^) + (0.0318*P*)^2^ + 1.4614*P*] where *P* = (*F*_o_^2^ + 2*F*_c_^2^)/3
4771 reflections	(Δ/σ)_max_ = 0.001
225 parameters	Δρ_max_ = 0.65 e Å^−^^3^
0 restraints	Δρ_min_ = −0.34 e Å^−^^3^

### Special details

**Table:**

Experimental. Crystal decay was monitored by repeating the initial 50 frames at the end of the data collection and analyzing duplicate reflectionsNMR data (p.p.m., CDCl_3_): ^1^H NMR, 0.88 (m, 6H, PCH_2_C*H*_3_), 1.83 (q, ^3^*J*(^1^H-^1^H) = 7 Hz, 4H, PC*H*_2_CH_3_), 2.24 (s, 6H, 2,4,6-*Me*_3_C_6_H_2_), 2.33 (s, 12H, 2,4,6-*Me*_3_C_6_H_2_), 6.78 (s, 4H, 2,4,6-Me_3_C_6_*H*_2_); ^13^C{^1^H}11.7 (PCH_2_*C*H_3_), 13.8 (P*C*H_2_CH_3_), 21.3 (s, 2,4,6-*Me*_3_C_6_H_2_), 27.7 (s, 2,4,6-*Me*_3_C_6_H_2_), 126.8 (s, 2,4,6-Me_3_*C*_6_H_2_), 136.6 (s, 2,4,6-Me_3_*C*_6_H_2_), 144.1 (s, 2,4,6-Me_3_*C*_6_H_2_); ^31^P NMR, *δ* -44.63 (*s*). FT—IR: 538*m*, 607vw, 673w, 708w, 752w, 800w, 845*m*, 976w, 1030w, 1042w, 1090w, 1149vw, 1244w, 1259w, 1288w, 1402w, 1547w, 1595vw, 1712vw.
Geometry. All e.s.d.'s (except the e.s.d. in the dihedral angle between two l.s. planes) are estimated using the full covariance matrix. The cell e.s.d.'s are taken into account individually in the estimation of e.s.d.'s in distances, angles and torsion angles; correlations between e.s.d.'s in cell parameters are only used when they are defined by crystal symmetry. An approximate (isotropic) treatment of cell e.s.d.'s is used for estimating e.s.d.'s involving l.s. planes.
Refinement. Refinement of *F*^2^ against ALL reflections. The weighted *R*-factor *wR* and goodness of fit *S* are based on *F*^2^, conventional *R*-factors *R* are based on *F*, with *F* set to zero for negative *F*^2^. The threshold expression of *F*^2^ > σ(*F*^2^) is used only for calculating *R*-factors(gt) *etc*. and is not relevant to the choice of reflections for refinement. *R*-factors based on *F*^2^ are statistically about twice as large as those based on *F*, and *R*-factors based on ALL data will be even larger.

### Fractional atomic coordinates and isotropic or equivalent isotropic displacement parameters (Å^2^)

**Table:**

	*x*	*y*	*z*	*U*_iso_*/*U*_eq_	
In1	0.231901 (11)	0.163644 (15)	0.585903 (16)	0.03479 (8)	
P1	0.21902 (4)	0.33051 (6)	0.54684 (6)	0.03532 (19)	
C1	0.14507 (16)	0.0724 (2)	0.5087 (2)	0.0355 (7)	
C2	0.07490 (17)	0.0952 (2)	0.4400 (2)	0.0430 (8)	
C3	0.02405 (18)	0.0319 (3)	0.3942 (3)	0.0504 (9)	
H3	−0.0230	0.0488	0.3476	0.061*	
C4	0.03963 (19)	−0.0550 (3)	0.4142 (3)	0.0512 (10)	
C5	0.10908 (19)	−0.0781 (2)	0.4811 (3)	0.0466 (9)	
H5	0.1210	−0.1375	0.4954	0.056*	
C6	0.16177 (17)	−0.0161 (2)	0.5276 (2)	0.0381 (8)	
C7	0.0530 (2)	0.1886 (3)	0.4137 (3)	0.0607 (11)	
H7A	0.0020	0.1922	0.3735	0.091*	
H7B	0.0710	0.2124	0.3695	0.091*	
H7C	0.0716	0.2216	0.4812	0.091*	
C8	−0.0172 (2)	−0.1223 (3)	0.3645 (4)	0.0719 (13)	
H8A	−0.0539	−0.1079	0.3803	0.108*	
H8B	0.0023	−0.1790	0.3951	0.108*	
H8C	−0.0369	−0.1236	0.2858	0.108*	
C9	0.23652 (17)	−0.0451 (2)	0.5954 (3)	0.0464 (8)	
H9A	0.2378	−0.1068	0.6114	0.070*	
H9B	0.2595	−0.0124	0.6634	0.070*	
H9C	0.2607	−0.0350	0.5553	0.070*	
C10	0.30035 (17)	0.1564 (2)	0.7643 (2)	0.0394 (8)	
C11	0.37154 (17)	0.1357 (2)	0.8185 (2)	0.0427 (8)	
C12	0.41094 (19)	0.1333 (2)	0.9321 (3)	0.0512 (9)	
H12	0.4590	0.1193	0.9670	0.061*	
C13	0.3812 (2)	0.1508 (2)	0.9945 (3)	0.0541 (10)	
C14	0.3113 (2)	0.1726 (2)	0.9417 (3)	0.0520 (10)	
H14	0.2903	0.1858	0.9834	0.062*	
C15	0.27101 (18)	0.1755 (2)	0.8287 (3)	0.0437 (8)	
C16	0.40779 (19)	0.1146 (3)	0.7567 (3)	0.0576 (10)	
H16A	0.3855	0.0644	0.7102	0.086*	
H16B	0.4569	0.1016	0.8080	0.086*	
H16C	0.4046	0.1642	0.7119	0.086*	
C17	0.4242 (2)	0.1459 (3)	1.1178 (3)	0.0770 (14)	
H17A	0.4729	0.1341	1.1407	0.115*	
H17B	0.4062	0.0994	1.1433	0.115*	
H17C	0.4210	0.2009	1.1488	0.115*	
C18	0.19456 (18)	0.1971 (3)	0.7786 (3)	0.0541 (10)	
H18A	0.1883	0.2598	0.7698	0.081*	
H18B	0.1779	0.1764	0.8260	0.081*	
H18C	0.1680	0.1692	0.7076	0.081*	
C19	0.29184 (17)	0.3701 (2)	0.6794 (2)	0.0414 (8)	
H19A	0.2829	0.3512	0.7371	0.050*	
H19B	0.3352	0.3416	0.6930	0.050*	
C20	0.30422 (19)	0.4667 (2)	0.6901 (3)	0.0496 (9)	
H20A	0.3211	0.4852	0.6421	0.074*	
H20B	0.3390	0.4808	0.7653	0.074*	
H20C	0.2605	0.4965	0.6698	0.074*	
C21	0.14350 (17)	0.3982 (2)	0.5211 (3)	0.0441 (8)	
H21A	0.1544	0.4590	0.5144	0.053*	
H21B	0.1031	0.3809	0.4505	0.053*	
C22	0.12205 (18)	0.3937 (3)	0.6068 (3)	0.0527 (9)	
H22A	0.1039	0.3360	0.6063	0.079*	
H22B	0.0859	0.4369	0.5905	0.079*	
H22C	0.1626	0.4053	0.6785	0.079*	

### Atomic displacement parameters (Å^2^)

**Table:**

	*U*^11^	*U*^22^	*U*^33^	*U*^12^	*U*^13^	*U*^23^
In1	0.02880 (13)	0.04999 (15)	0.01987 (11)	−0.01078 (11)	0.00826 (9)	0.00045 (10)
P1	0.0310 (4)	0.0489 (5)	0.0237 (4)	−0.0081 (4)	0.0122 (3)	−0.0014 (4)
C1	0.0328 (17)	0.053 (2)	0.0194 (14)	−0.0126 (15)	0.0121 (13)	−0.0020 (14)
C2	0.0355 (19)	0.059 (2)	0.0300 (17)	−0.0130 (17)	0.0137 (15)	−0.0048 (16)
C3	0.0330 (19)	0.070 (3)	0.043 (2)	−0.0120 (18)	0.0159 (16)	−0.0112 (18)
C4	0.042 (2)	0.071 (3)	0.046 (2)	−0.026 (2)	0.0267 (18)	−0.0234 (19)
C5	0.057 (2)	0.050 (2)	0.045 (2)	−0.0126 (18)	0.0349 (19)	−0.0124 (16)
C6	0.0377 (18)	0.054 (2)	0.0261 (15)	−0.0101 (16)	0.0188 (14)	−0.0049 (14)
C7	0.038 (2)	0.068 (3)	0.052 (2)	−0.0092 (19)	0.0052 (18)	0.0079 (19)
C8	0.061 (3)	0.085 (3)	0.077 (3)	−0.038 (2)	0.040 (2)	−0.035 (3)
C9	0.045 (2)	0.053 (2)	0.0365 (19)	−0.0034 (18)	0.0176 (17)	0.0005 (16)
C10	0.0374 (18)	0.050 (2)	0.0220 (15)	−0.0155 (16)	0.0089 (13)	0.0015 (14)
C11	0.0372 (19)	0.057 (2)	0.0255 (16)	−0.0129 (16)	0.0098 (14)	0.0013 (14)
C12	0.038 (2)	0.068 (2)	0.0303 (18)	−0.0126 (18)	0.0045 (15)	0.0032 (16)
C13	0.056 (2)	0.069 (3)	0.0236 (17)	−0.021 (2)	0.0109 (16)	0.0005 (16)
C14	0.053 (2)	0.072 (3)	0.0273 (17)	−0.018 (2)	0.0175 (16)	−0.0053 (17)
C15	0.0394 (19)	0.058 (2)	0.0277 (16)	−0.0146 (17)	0.0128 (14)	0.0006 (15)
C16	0.040 (2)	0.091 (3)	0.0348 (19)	−0.004 (2)	0.0141 (17)	0.002 (2)
C17	0.072 (3)	0.112 (4)	0.0230 (18)	−0.021 (3)	0.0070 (18)	0.002 (2)
C18	0.044 (2)	0.080 (3)	0.039 (2)	−0.007 (2)	0.0216 (18)	0.0019 (18)
C19	0.0381 (19)	0.058 (2)	0.0238 (16)	−0.0072 (16)	0.0126 (14)	−0.0021 (14)
C20	0.055 (2)	0.060 (2)	0.0350 (19)	−0.0194 (19)	0.0234 (17)	−0.0112 (16)
C21	0.0364 (19)	0.057 (2)	0.0365 (18)	−0.0003 (17)	0.0165 (15)	0.0003 (16)
C22	0.041 (2)	0.078 (3)	0.0373 (19)	−0.004 (2)	0.0193 (17)	−0.0078 (18)

### Geometric parameters (Å, °)

**Table:**

In1—C1	2.190 (3)	C11—C16	1.509 (5)
In1—C10	2.215 (3)	C12—C13	1.385 (5)
In1—P1	2.6300 (12)	C12—H12	0.9500
In1—P1^i^	2.6364 (9)	C13—C14	1.386 (5)
P1—C21	1.856 (3)	C13—C17	1.524 (5)
P1—C19	1.866 (3)	C14—C15	1.397 (4)
P1—In1^i^	2.6364 (9)	C14—H14	0.9500
C1—C2	1.406 (4)	C15—C18	1.514 (5)
C1—C6	1.409 (5)	C16—H16A	0.9800
C2—C3	1.386 (5)	C16—H16B	0.9800
C2—C7	1.512 (5)	C16—H16C	0.9800
C3—C4	1.384 (5)	C17—H17A	0.9800
C3—H3	0.9500	C17—H17B	0.9800
C4—C5	1.392 (5)	C17—H17C	0.9800
C4—C8	1.511 (5)	C18—H18A	0.9800
C5—C6	1.398 (4)	C18—H18B	0.9800
C5—H5	0.9500	C18—H18C	0.9800
C6—C9	1.510 (4)	C19—C20	1.515 (5)
C7—H7A	0.9800	C19—H19A	0.9900
C7—H7B	0.9800	C19—H19B	0.9900
C7—H7C	0.9800	C20—H20A	0.9800
C8—H8A	0.9800	C20—H20B	0.9800
C8—H8B	0.9800	C20—H20C	0.9800
C8—H8C	0.9800	C21—C22	1.530 (4)
C9—H9A	0.9800	C21—H21A	0.9900
C9—H9B	0.9800	C21—H21B	0.9900
C9—H9C	0.9800	C22—H22A	0.9800
C10—C11	1.405 (5)	C22—H22B	0.9800
C10—C15	1.408 (5)	C22—H22C	0.9800
C11—C12	1.402 (4)		
			
C1—In1—C10	117.60 (11)	C13—C12—C11	121.4 (3)
C1—In1—P1	123.68 (9)	C13—C12—H12	119.3
C10—In1—P1	103.40 (9)	C11—C12—H12	119.3
C1—In1—P1^i^	104.11 (7)	C12—C13—C14	118.2 (3)
C10—In1—P1^i^	122.61 (9)	C12—C13—C17	120.9 (4)
P1—In1—P1^i^	81.56 (3)	C14—C13—C17	120.9 (4)
C21—P1—C19	104.40 (16)	C13—C14—C15	121.4 (3)
C21—P1—In1	125.97 (11)	C13—C14—H14	119.3
C19—P1—In1	99.36 (11)	C15—C14—H14	119.3
C21—P1—In1^i^	120.44 (11)	C14—C15—C10	120.9 (3)
C19—P1—In1^i^	104.85 (10)	C14—C15—C18	117.5 (3)
In1—P1—In1^i^	98.44 (3)	C10—C15—C18	121.6 (3)
C2—C1—C6	118.1 (3)	C11—C16—H16A	109.5
C2—C1—In1	125.1 (2)	C11—C16—H16B	109.5
C6—C1—In1	116.8 (2)	H16A—C16—H16B	109.5
C3—C2—C1	120.3 (3)	C11—C16—H16C	109.5
C3—C2—C7	118.4 (3)	H16A—C16—H16C	109.5
C1—C2—C7	121.3 (3)	H16B—C16—H16C	109.5
C4—C3—C2	122.1 (3)	C13—C17—H17A	109.5
C4—C3—H3	119.0	C13—C17—H17B	109.5
C2—C3—H3	119.0	H17A—C17—H17B	109.5
C3—C4—C5	117.9 (3)	C13—C17—H17C	109.5
C3—C4—C8	120.8 (4)	H17A—C17—H17C	109.5
C5—C4—C8	121.3 (4)	H17B—C17—H17C	109.5
C4—C5—C6	121.5 (3)	C15—C18—H18A	109.5
C4—C5—H5	119.3	C15—C18—H18B	109.5
C6—C5—H5	119.3	H18A—C18—H18B	109.5
C5—C6—C1	120.1 (3)	C15—C18—H18C	109.5
C5—C6—C9	119.1 (3)	H18A—C18—H18C	109.5
C1—C6—C9	120.8 (3)	H18B—C18—H18C	109.5
C2—C7—H7A	109.5	C20—C19—P1	116.6 (2)
C2—C7—H7B	109.5	C20—C19—H19A	108.1
H7A—C7—H7B	109.5	P1—C19—H19A	108.1
C2—C7—H7C	109.5	C20—C19—H19B	108.1
H7A—C7—H7C	109.5	P1—C19—H19B	108.1
H7B—C7—H7C	109.5	H19A—C19—H19B	107.3
C4—C8—H8A	109.5	C19—C20—H20A	109.5
C4—C8—H8B	109.5	C19—C20—H20B	109.5
H8A—C8—H8B	109.5	H20A—C20—H20B	109.5
C4—C8—H8C	109.5	C19—C20—H20C	109.5
H8A—C8—H8C	109.5	H20A—C20—H20C	109.5
H8B—C8—H8C	109.5	H20B—C20—H20C	109.5
C6—C9—H9A	109.5	C22—C21—P1	116.1 (2)
C6—C9—H9B	109.5	C22—C21—H21A	108.3
H9A—C9—H9B	109.5	P1—C21—H21A	108.3
C6—C9—H9C	109.5	C22—C21—H21B	108.3
H9A—C9—H9C	109.5	P1—C21—H21B	108.3
H9B—C9—H9C	109.5	H21A—C21—H21B	107.4
C11—C10—C15	117.3 (3)	C21—C22—H22A	109.5
C11—C10—In1	124.8 (2)	C21—C22—H22B	109.5
C15—C10—In1	117.9 (2)	H22A—C22—H22B	109.5
C12—C11—C10	120.8 (3)	C21—C22—H22C	109.5
C12—C11—C16	118.0 (3)	H22A—C22—H22C	109.5
C10—C11—C16	121.2 (3)	H22B—C22—H22C	109.5

Symmetry codes: (i) −*x*+1/2, −*y*+1/2, −*z*+1.
